# A Flexible Proximity Sensor Fully Fabricated by Inkjet Printing

**DOI:** 10.3390/s100505054

**Published:** 2010-05-19

**Authors:** Chin-Tsan Wang, Kuo-Yi Huang, David T. W. Lin, Wei-Chia Liao, Hua-Wei Lin, Yuh-Chung Hu

**Affiliations:** 1 Department of Mechanical and Electromechanical Engineering, National ILan University, ILan 260, Taiwan; E-Mails: ctwang@niu.edu.tw (C.-T.W.); m9622008@cat.hfu.edu.tw (W.-C.L.); r9722013@ms.niu.edu.tw (H.-W.L.); 2 Department of Mechatronic Engineering, Huafan University, Taipei 223, Taiwan; E-Mail: kyhuang@huafan.hfu.edu.tw; 3 Graduate Institute of Mechatronic System Engineering, National University of Tainan, Tainan 70005, Taiwan; E-Mail: david@mail.nutn.edu.tw

**Keywords:** flexible electronics, inkjet printing, proximity sensor, pyroelectric

## Abstract

A flexible proximity sensor fully fabricated by inkjet printing is proposed in this paper. The flexible proximity sensor is composed of a ZnO layer sandwiched in between a flexible aluminum sheet and a web-shaped top electrode layer. The flexible aluminum sheet serves as the bottom electrode. The material of the top electrode layer is nano silver. Both the ZnO and top electrode layers are deposited by inkjet printing. The fully inkjet printing process possesses the advantages of direct patterning and low-cost. It does not require photolithography and etching processes since the pattern is directly printed on the flexible aluminum sheet. The prototype demonstrates that the presented flexible sensor is sensitive to the human body. It may be applied to proximity sensing or thermal eradiation sensing.

## 1. Introduction

Flexible sensors have emerging applications in biomedicine, artificial skin, and wearable electronics [1]. One of the ways to make the sensor flexible is to fabricate the sensor device or circuit directly on flexible substrates. This is exactly the way adopted in this work because it is the most direct and innovative approach to the manufacturing of large-area flexible electronic devices. Pyroelectric materials have been gradually applied in thermal detectors. Numerous aspects of our daily lives make use of thermal detection, such as automatic flushing apparatuses in toilets, automatic doors and lights …*etc*. In biomedicine applications, implanted chips with micro temperature sensors could be used to detect the abnormal cells, such as cancer cells, since the temperatures of abnormal cells are higher than those of the normal ones. In the textile industry, flexible electronics are embedded into the fibers or clothes to make clothing with the ability to monitor biological aspects. ZnO is a unique material that exhibits the multiple properties of semiconductivity, piezoelectricity, and pyroelectricity. The pyroelectricity of ZnO is characterized by its non-centrosymmetrical crystals with a specific polar axis along the direction of spontaneous polarization. The internal polarization of ZnO produces an electric field as it is subjected to temperature variations. Thin film pyroelectric sensors possess the advantages of being integrable with integrated circuits (IC), un-cooled detecting, operation at room temperature, fast and wide spectral responses, high sensitivity, and low cost [2,3]. There are many methods for the preparation of ZnO films, such as DC or RF sputtering [4], chemical vapor deposition [5], metal organic chemical vapor deposition [6], sol-gel method [7], and spray pyrolysis [8]. Among the aforementioned methods the sol-gel method is low cost and it allows the production of large-area films with good homogeneity and the chemical ingredients and concentration are also easy to control. For these reasons the sol-gel method had been applied in ZnO thin film deposition processes. The intrinsic characteristic dimensions of flexible electronics are typically on a scale of several tens of micrometers. From the cost-benefit perspective, if flexible sensors could be fabricated by printing, their cost could be reduced to a few dollars per square meter [9]. Thanks to the advancements of material science, many thin-film materials, such as noble-metal conductors, organic conductors, semiconductors, and insulators, can be prepared nowadays by inkjet printing. Furthermore, flexible substrates cannot sustain the high temperatures of IC fabrication processes. Inkjet printing is a high-throughput process, roll-to-roll process compatible, may not require vacuum, and may provide a solution to overlay registration problems through digital compensation. Printing metallic conductors from nanoparticles may reduce the required sintering temperature to values acceptable for plastic substrates. Inkjet printing uses device materials more efficient than screen printing. These are the reasons why inkjet printing technology has become a hot process technology for flexible electronics.

Thermal detectors can be classified into two types: one is the thermal type and the other one is the photon type. The photon type can further classified into photo-conductive and photo-voltaic types. The thermal type contains the thermopile, thermo-resist, and pyroelectric types. In general photon type detectors have the advantages of faster response and high sensitivity, but also the disadvantages of complicated structures, low operation temperature (lower than room temperature), and high cost. The thermal type detectors have the advantages of low cost, simple structures, and operation at room temperature while their main disadvantage is a slower response. This work proposes a flexible proximity sensor fully fabricated by inkjet printing. The prototype successfully demonstrates sensitivity to the human body and may be applied in situations requiring thermal detection.

## 2. The Sensor Structure

**Figure 1.**
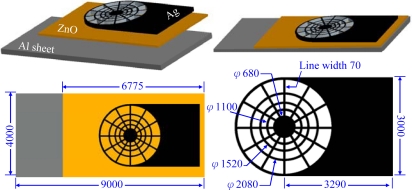
The senor structures and dimensions in μm.

The proposed flexible proximity sensor is composed of a ZnO layer sandwiched between a flexible aluminum sheet and a web-shaped top electrode layer, as shown in Figure 1. The flexible aluminum sheet serves as the bottom electrode. The thickness of the flexible aluminum sheet is 13 μm. The web-shaped top electrode layer is made of nano silver. The temperature change of the ZnO layer will induce an electric potential between the top and bottom electrodes due to the internal polarization of ZnO. The pyroelectric signal is proportional to the temperature variation rate throughout the ZnO layer. In other words, a higher and more uniform temperature variation rate throughout the ZnO layer leads to a higher response of the pyroelectric sensor. Partially covered top-electrodes have shown higher responsivity than that of fully covered top-electrodee because the uncovered part of the ZnO layer is directly exposed to the heat source and thus the heat absorption is greatly increased [3]. The responsivity is defined as the ratio of the output voltage of the sensor to the incident power. The authors designed a web-shaped top electrode which not only improves the heat absorption but also the heat uniformity throughout the ZnO layer. The responsivity of the sensor may be improved by opening the windows so that the ZnO layer can come into direct contact with the heat source. On the other hand, the contact windows may reduce the top-electrode area and disperse the electrode and this electrode area reduction and dispersion may degrade the responsivity of the sensor. Thus, in the layout design of the top electrode, both the size of the ZnO layer contact window and the dispersion of the top electrodes must be considered. This work designs a web-type top electrode. The outer regions of the web type possess large ZnO layer contact windows, whereas the inner regions possess dense top electrodes.

## 3. Preparation of Inkjet Solutions

**Figure 2.**
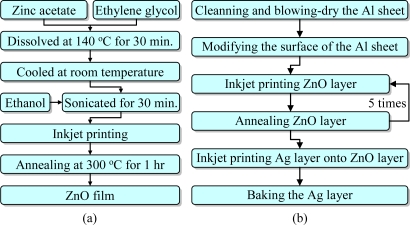
(a) The preparation process of ZnO inkjet solution; (b) the fabrication process flow of the flexible pyroelectric sensor.

The ZnO inkjet solution is prepared by the sol-gel method, as shown in Figure 2(a). It is synthesized by dissolving 0.4 mol of zinc acetate into 120 mL ethylene glycol and heating at 140 °C for 30 min, which results in a transparent solution. However, the solution will settle after cooling at room temperature for about 30 minutes. Therefore 280 mL ethanol are add into the solution which is sonicated for 30 minutes until the sediment is completely dissolved in ethanol. Filtration of the solution affords a clear and homogeneous ZnO solution. Its molal concentration is 1 M, its viscosity is 14 CPS, its surface tension is 30 dynes/cm, and its pH value is 6. The inkjet material of the top electrode layer is a commercial alcohol-based nano-silver ink provided by Cabot Conductive Ink (CCI-300). Its viscosity and surface tension at room temperature are about 11–15 CPS and 30–33 mN/m respectively, the silver solids loading is about 19–21 wt%, and its density is about 19–21 g/mL.

## 4. Fabrication Process

**Figure 3.**
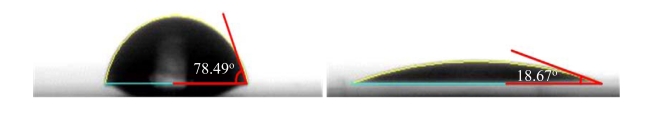
The contact angle of the aluminum sheet before and after plasma modification.

The overall process flow is shown in Figure 2(b) while the parameters are detailed in Table 1. Firstly, the flexible aluminum sheet is cleaned with ethanol and then blow-dried with nitrogen. Then atmospheric plasma is used to make the surface of the aluminum sheet more hydrophilic, to improve the adhesion of the ZnO solution on the aluminum sheet. The aluminum sheet is placed onto the stage of the plasma equipment. The plasma is sprayed by a nozzle whose orifice diameter is 4 mm. The orifice of the plasma spray nozzle is at a distance of 25 mm from the aluminum sheet. Figure 3 shows that the contact angle changes from 78.49° to 18.67° after the plasma modification.

**Figure 4.**
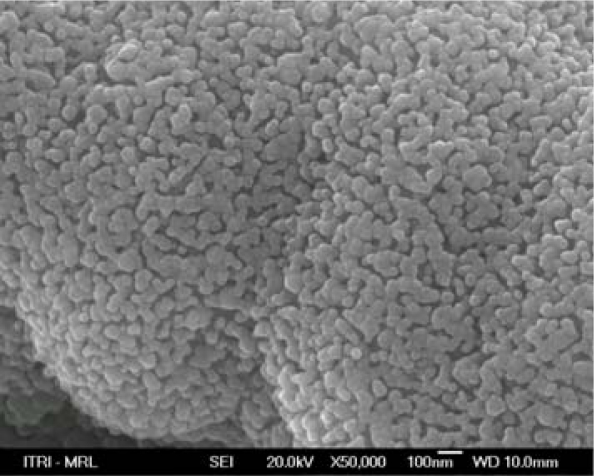
SEM photo of the ZnO layer.

Subsequently, a Dimatix materials printer (DMP-2800) is used the print the ZnO solution onto the Al sheet. The inkjet printing parameters are detailed in Table 1. To make a better ZnO film, it must be annealed in an oven right after the inkjet printing process. The aforementioned two steps, *i.e*., ZnO inkjet printing and annealing, are repeated five times, giving a 5 μm ZnO film. Figure 4 shows the morphology of the ZnO film visualized with the aid of a scanning electron microscope (SEM).

**Figure 5.**
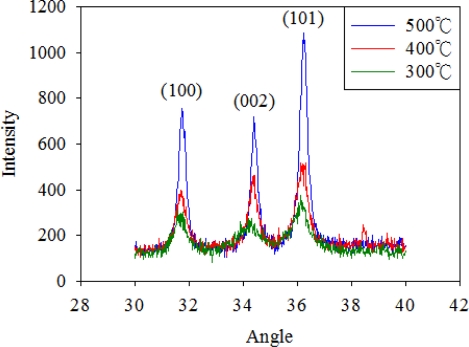
The XRD spectra of ZnO layer at different anneal temperature.

**Figure 6.**
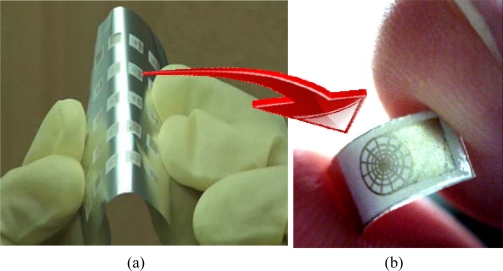
The flexible proximity senor elements: (a) a batch of flexible proximity sensors which are batch-manufactured on a flexile aluminum sheet; (b) a single flexible proximity sensor which is cut from (a).

Figure 5 shows the X-ray diffraction (XRD) spectra of the ZnO films at different annealing temperatures, namely 300, 400, and 500 °C. The peaks at 31.88°, 34.58° and 36.38° are due to the diffraction from the (100), (002) and (101) crystalline plane of the quartzite structure of ZnO respectively, according to the peaks of standard ZnO (DBCard number: 01-075-6445). From the XRD measurement results, one could conclude that the lattice intensity increases as the annealing temperature increases. The pyroelectricity of ZnO is characterized by non-centro-symmetrical crystals and has a specific polar axis along the direction of spontaneous polarization. The internal polarization of ZnO will produces an electric field as it is subjected to temperature variation. Therefore, the lattice intensity will affect the pyroelectricity of ZnO and thus affect the sensor performance. However, the melting point of aluminum is about 660 °C. Therefore, this work anneals the ZnO film at 500 °C. The last step is to print the conductive nano-silver which serves as the top electrode. The inkjet printing parameters are detailed in Table 1. Figure 6 shows the finished flexible proximity sensor element.

## 5. Signal Measurement

**Figure 7.**
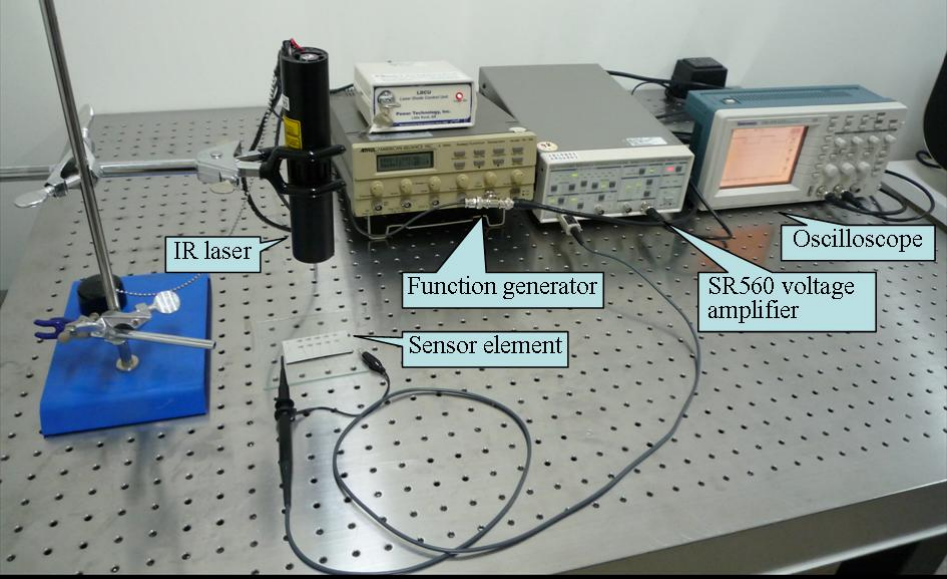
The signal measurement setup.

Figure 7 shows the signal measurement setup. The radiation source is an infrared (IR) laser. Its wavelength and maximum power are 905 nm and 7 mW, respectively. The wave form and frequency of the IR ray are modulated by a function generator. The modulated IR ray irradiates the proximity sensor. The output signal of the proximity sensor is amplified by the SR560 low-noise voltage amplifier and then displayed on a digital oscilloscope.

**Figure 8.**
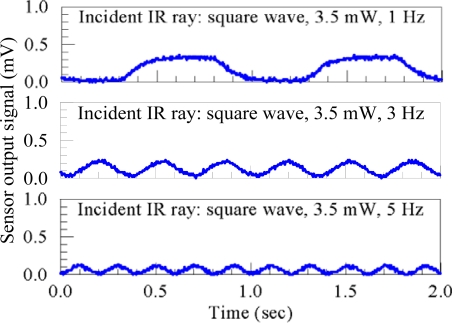
The measurement signals in the time domain.

Figure 8 shows some measured signals in the time domain. It should be mentioned here that the signals shown in Figure 8 are the original signal output of the proximity sensor, that is, without amplification by the SR560 amplifier.

**Figure 9.**
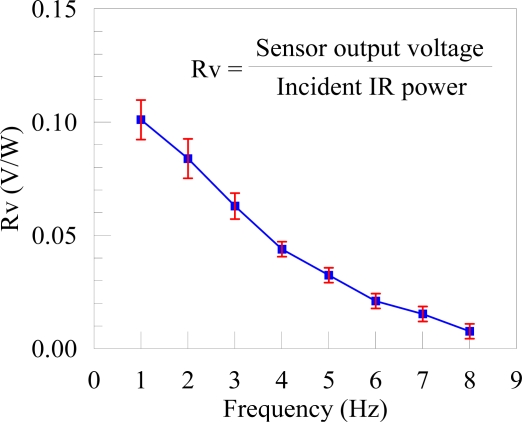
The sensitivities of the sensor to the frequencies of incident IR ray.

Figure 9 shows the sensitivities (Rv) of the present sensor to the frequencies of the incident IR ray. The value of Rv is obtained from the ratio of the original signal output by the proximity sensor to the power of the incident IR ray. The error bars shown in Figure 9 are obtained from three batches of the sensors samples. A batch, as shown in Figure 6(a), contains 25 sensors. Define the signal to noise ratio (SNR) as SNR = 20 log_10_(*V_S_*/*V_N_*) dB in which *V_S_* is the sensor output signal in mV and the noise level *V_N_* also in mV. The average SNR of the present flexible proximity sensor is about 50 dB.

## 6. Prototype Demonstration

**Figure 10.**
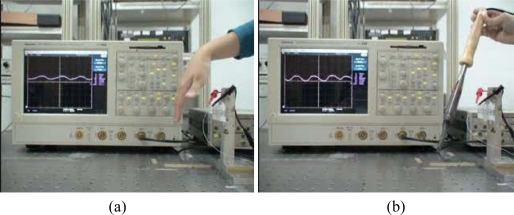
A demonstration of the flexible proximity sensor: A corresponding signal appears on the oscilloscope when (a) a shaking hand and (b) a hot welding gun approach the sensor.

The outputs of the flexible proximity sensor were connected to an oscilloscope, as shown in Figure 10. One can observes that the corresponding signals output by the sensor are shown on the oscilloscope when (a) a shaking hand and (b) a shaking hot welding gun approach the sensor. Figure 10 (b) verifies that the sensor output signal really comes from the hot irradiation of bodies but not electrostatic field because the hot welding gun is a closed loop circuit. The output signal becomes larger as the shaking hand get closer to the sensor. The prototype demonstration shows that the proposed flexible sensor can be applied to human body proximity sensing.

## 7. Conclusions

The proposed flexible proximity sensor successfully demonstrated the detection of a hot object. The average signal to noise ratio is high (50 dB). The fully inkjet printing process possesses the advantage of direct and fast layout patterning. This is a major breakthrough that substantially reduces the fabrication process and cost. The flexible proximity sensor may also be used in artificial skin for robots or wearable sensing devices, being integrable with clothes to sense the environmental temperature variation or elsewhere requiring proximity sensing of hot bodies.

## Figures and Tables

**Table 1.** Recipes for the sensor fabrication process.

Plasma	Gas	Compressed dry air
	Flow rate	45 slm
	Power	350 W/20 kHz
	Stage moving speed	10 mm/s in x-direction
	Stage moving pitch	2 mm in y-direction

Inkjet printing	Firing voltage	28 V
	Drop space	30 μm
	Jetting frequency	1 kHz
	Substrate temperature	60 °C
	Cartridge print height	1 mm

ZnO anneal	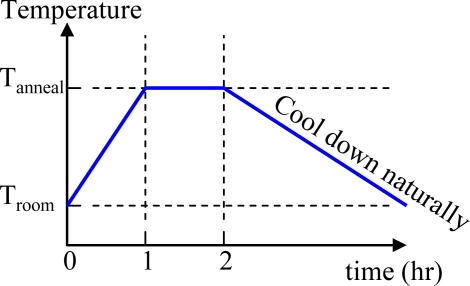	

Nano-silver curing	Oven Vacuum	6.7×10^−2^ Pa
	Temperature	200 °C
	Time	30 min.
